# Job loss and psychological distress during the COVID-19 pandemic: a national prospective cohort study

**DOI:** 10.1186/s12889-023-16303-5

**Published:** 2023-07-28

**Authors:** Jonathan Wörn, Bjørn-Atle Reme, Vegard Skirbekk

**Affiliations:** Center for Fertility and Health, Norwegian Institute of Public Health, Postbox 222 Skøyen, N-0213 Oslo, Norway

**Keywords:** Job loss, Mental health, COVID-19, Social inequality

## Abstract

**Background:**

The COVID-19 pandemic caused substantial increases in unemployment; however, the association between these job losses and psychological distress is not well documented. Our study reports on this association from a cohort study, with a particular focus on educational differences in both the likelihood of job loss and its potential implications for mental health.

**Methods:**

Utilizing data from a large prospective cohort study of parents in Norway (*n* = 58,982), we examined changes in psychological distress within four groups of respondents: those who during the first wave of COVID-19 had (i) no change in their employment situation, (ii) worked from home, (iii) been furloughed, or (iv) lost their job.

**Results:**

Psychological distress increased in all groups. In z-scores relative to pre-pandemic levels, the increases were (i) 0.47 [95%-CI: 0.45–0.49] among respondents with no change in their employment situation, (ii) 0.51 [95%-CI: 0.49–0.53] among respondents who worked from home, (iii) 0.95 [95%-CI:0.91–0.99] among those furloughed, and (iv) 1.38 [95%-CI: 1.16–1.59] among those who permanently lost their job, corresponding to increases of 89%, 95%, 170%, and 185%, respectively. While respondents without university education had a 2 to 3 times higher risk of job loss, the negative impact of job loss on psychological distress was similar across educational levels.

**Conclusions:**

Participants exposed to job loss during the pandemic experienced a stronger increase in symptoms of depression or anxiety compared to those who remained employed. Although higher education lowered the risk of losing work, it did not substantially diminish the impact on mental health from losing work.

**Supplementary Information:**

The online version contains supplementary material available at 10.1186/s12889-023-16303-5.

## Background

The governmental restrictions during the COVID-19 pandemic affected the mental health, substance use and general well-being of many persons around the world [1–4]. It also widely affected employment, with many working from home, being on temporary furlough, or confronted with job loss. These effects were not equally distributed. For example, young male workers with low levels of education experienced a stronger increase in the risk of job loss [5]. While the differences in employment effects across socioeconomic position have been documented, less is known about the impacts on mental health.

The literature on the potential implications of job loss on health finds effect sizes varying from substantial [6–8] to quite limited [9]. Moreover, the evidence suggests that the wider economic context in which job loss occurs matters for how strong the effects are [7, 10]. The pandemic was in several respects vastly different from previous downturns, suggesting that previous research to a limited extent is informative of the impacts from job loss during COVID-19 on mental health.

Therefore, our study contributes to the small, but rapidly growing, literature documenting the relationship between employment experiences during the COVID-19 pandemic and mental health [4, 11–15]. These studies find higher levels of depression or anxiety among individuals experiencing income losses or threats to their personal finances [4, 14], higher job insecurity [11] or job loss [11–13, 15]. Although the evidence so far suggests a negative relationship, the studies have limited sample sizes and are cross-sectional. Studies using large longitudinal data and validated instruments to compare mental health before and during the pandemic for persons with different employment experiences and levels of education are scarce [16].

Against this background, the overall aim of this study was to assess the impact of job loss on psychological distress during the first wave of the COVID-19-pandemic in spring 2020, with particular attention to educational differences and gender differences in both the likelihood of job loss and its implications for mental health.

## Methods

### Design

The study was based on the Norwegian Mother, Father and Child Cohort Study (MoBa). MoBa is a population-based pregnancy cohort study conducted by the Norwegian Institute of Public Health [17]. In total, it includes approximately 95,000 mothers and 75,000 fathers. The MoBa participants were recruited from all over Norway between 1999 and 2008 and followed-up thereafter. The first MoBa-questionnaire was administered to female participants in the 15th week of pregnancy, with ten follow-up surveys at specific child ages until the child was 14 years old, but no later than 2018. Fathers were surveyed twice: in the 15th week of pregnancy, and once in the period 2015-2017. After the introduction of measures to mitigate the spread of SARS-Cov2 in Norway in March 2020, these regular MoBa-interviews were supplemented with additional interviews aiming at assessing the health consequences of SARS-Cov2 in the cohort. Approximately 150,000 MoBa-participants were invited to participate in data collections beginning in March 31, 2020, with interviews recurring every 14 days thereafter for an extended period of time.

### Participants

This study included mothers and fathers who participated in the MoBa-study. Our study sample was restricted to participants who responded to questions about mental health at least once before, and at least once during the pandemic (*n* = 58,982) and did not have missing values on relevant variables.

### Measures

This study uses the *pre-pandemic* mental health measures of mothers from the assessments in the 15th week of pregnancy, and from when the child was five and eight years old. For fathers, both available pre-pandemic data collections were used. *During* the pandemic, we used the first three waves of the SARS-Cov2-related data collections between March 31 and May 12, 2020, which contain information on mental health and employment changes due to the COVID-19 pandemic. See Table S1 in the Supplementary material for an overview of the survey waves used.

#### Outcomes

The primary outcome was symptoms of depression or anxiety (referred to as “depressive symptoms” throughout the paper), measured with the five-item version of the Hopkins Symptom Checklist (SCL-5) [18, 19]. The items assess whether respondents have—during the past two weeks—been bothered by 1) feeling fearful, 2) nervousness or shakiness inside, 3) feeling hopeless about the future, 4) feeling blue, and 5) worrying too much about things. Response options included *not bothered (0), a little bothered (1), quite bothered (2),* and *very bothered (3)*. We analyse the mean value across the five items. Schmalbach et al. (2021) and Strand et al. (2003) assessed the psychometric properties of the SCL-5 and found high correlations (> 0.8) between the 5-items scale and the longer 25-items version [18, 20]. They also reported satisfactory reliability (α = 0.84 and α = 0.88, respectively) and theoretically expected correlations with gender, family, and socioeconomic status.

#### Exposures

The exposure was self-reported *change in job status due to COVID-19*, with the following four groups: *(i) no change, (ii) home office,*^1^* (iii) on furlough,* and *(iv) lost job.* These categories were constructed based on a question in the 2020 surveys (i.e., during the pandemic) about changes in employment due to COVID-19.^2^ From the 125,181 individuals that responded to at least one of the three relevant surveys conducted during the COVID-19 pandemic, we excluded individuals whose response about the employment situation differed across the three waves, responded with multiple employment statuses, or did not respond regarding employment status. Following these restrictions, we were left with 61,157 individuals with a measured exposure. Throughout the analysis, for ease of exposition, we refer to furlough as *temporary job loss* and lost job as *permanent job loss*. When we refer to *job loss* without any mention of its duration, we include both categories.

### Statistical analysis

#### Main analysis

**Fig.1:**
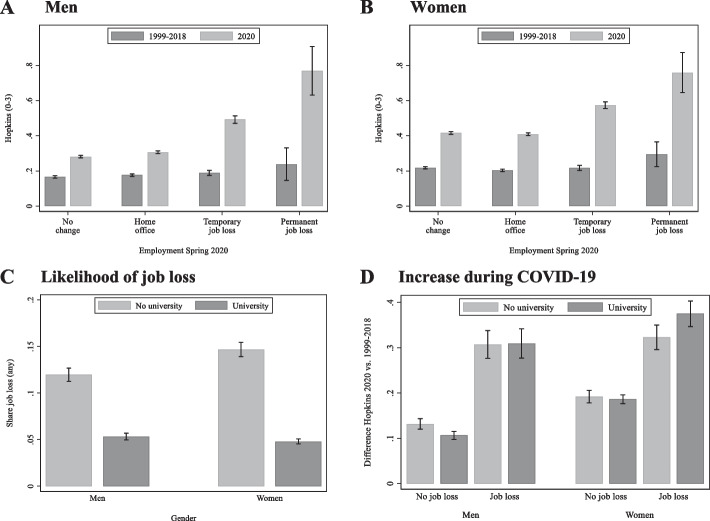
**A** and **B** Mean depressive symptoms (with 95%-confidence intervals) before and during the first wave of COVID-19 among participants who experienced home office, temporary job loss or permanent job loss, or no change in their job situation during the COVID-19 pandemic. Panel** A** men; Panel** B** women. ** C** Likelihood of job loss (temporary or permanent) during the first wave of COVID-19, by gender and education. ** D**: Change in depressive symptoms from before to during the first wave of COVID-19, by employment situation, gender, and education. All figures show predicted values from multivariate linear regressions (**A**, **B**, **D**) or logit regression (**C**). All models account for number of persons in the household and age of the respondent. No job loss = no change or home office; job loss = temporary job loss or permanent job loss

To characterise the association between change in employment status during the COVID-19 pandemic and change in psychological distress, we estimated a pooled linear regression model where depressive symptoms were regressed on dummy variables for each employment group (person-level variable, reference: *no change*), a dummy for the COVID-19 period (reference: before the COVID-19-pandemic), and their interaction (to assess differences in the pre-during change between the employment groups). The levels and standard errors reported in Fig. 1 were retrieved from the coefficients of this pooled regression model (see Tables S3 and S4 for details) using the *margins*-command in Stata.

#### Analysis of effect heterogeneities and exposure heterogeneities

We explored exposure and effect heterogeneities by education and gender by running additional models. First, related to exposure heterogeneities, we estimated the likelihood of job loss by educational level and gender. This was achieved by a logistic regression model with a binary indicator variable of job loss as the outcome, and education, gender, and their interaction as predictors (see Table S5). Second, related to effect heterogeneities, we estimated how the change in psychological distress following job loss varied across education levels and gender. For this, we used a linear regression model similar to the main analysis, but using a binary indicator of job loss (both temporary and permanent) to increase statistical power. In addition, a dummy for university education (vs. no university) was added. Furthermore, all relevant variables for the three-way interaction of job loss, time period, and education were included in the model. The graphical representation of these models in Fig. 1 panels C and D was also obtained using the *margins*-command in Stata. All models included dummy variables to control for age at interview as well as the number of other persons living in the household (categories: 0, 1, 2, 3, 4, 5 or more). The latter was operationalized as the maximum number of persons reported in the three data collections during the pandemic in 2021. All models account for standard error inflation arising from multiple observations from the same individual by using cluster-corrected inference statistics on the level of individuals. All analyses were conducted in Stata MP v.16.

## Results

Our analytical sample included 58,982 participants with non-missing information on relevant variables. Among the 57,433 individuals participating in the first COVID-19 interview in 2020, 54.5% were categorized as *no change in employment situation*, 37.7% as *home office*, 7.5% as *temporary job loss*, and 0.3% as *permanent job loss*. 59% of these participants were female, and the average age was 47.1 years (SD=5.2). 67% reported having a university degree.^3^ See Table S2 in the Supplementary material for more detailed descriptive statistics.

While there were only minor differences in depressive symptoms *before* the pandemic across employment groups, levels were clearly higher among those who *during* the pandemic lost work (Fig. 1, Panels A and B). The increase for men with no change in employment during the pandemic was 0.12 [95%-CI: 0.11-0.12] scale points, compared to 0.13 [95%-CI: 0.12-0.14] for home office, 0.30 [95%-CI: 0.28-0.33] for temporary job loss, and 0.53 [95%-CI: 0.40-0.66] for permanent job loss. Corresponding z-scores – generated from the pre-pandemic mean and standard deviation in the joint sample for men and women – are 0.32 [95%-CI: 0.29-0.34] for no change, 0.36 [95%-CI: 0.33-0.39] for home office, 0.84 [95%-CI: 0.78-0.90] for temporary job loss, and 1.46 [95%-CI: 1.11-1.82] for permanent job loss. Compared to their respective pre-pandemic levels, depressive symptoms increased by 65%, 72%, 221%, and 158% in the different employment groups.

Among women (Panel B), the increase for those with no change in employment was 0.20 [95%-CI: 0.19-0.21] scale points, compared to 0.21 [95%-CI: 0.20-0.22] for home office, 0.36 [95%-CI: 0.34-0.38] for temporary job loss, and 0.46 [95%-CI: 0.37-0.56] for permanent job loss. Corresponding z-scores are 0.55 [95%-CI: 0.52-0.58] for no change, 0.57 [95%-CI: 0.54-0.60] for home office, 0.99 [95%-CI: 0.93-1.04] for temporary job loss, and 1.29 [95%-CI: 1.02-1.54] for permanent job loss. Compared to their respective pre-pandemic levels, depressive symptoms increased by 91%, 105%, 153%, and 159%.

Among respondents without university education (Panel C), 12% [95%-CI: 11%-13%] of men and 15% [95%-CI: 14%-15%] of women reported job loss (*temporary* or *permanent* vs. *no change* or *home office*). In contrast, only 5% [95%-CI: 5%-5% (women), 5%-6% (men)] of respondents with a university education reported job loss. The odds ratios for job loss for those with university education (vs. no university) were 0.41 [95%-CI: 0.38-0.46] for men and 0.29 [95%-CI: 0.27-0.32] for women (see Table S5). For both job loss exposure groups, the increase in depressive symptoms during the pandemic was generally similar across educational levels (Panel D). Among those who did not experience job loss, the increase was between 0.11 and 0.13 scale points (men) and close to 0.19 scale points (women). Among those with job loss, the increase was close to 0.31 scale points (men) and between 0.32 and 0.38 scale points (women; Table S6 and S7). The slight difference in increase between women with and without university education was statistically significant (0.06 scale points, 95%-CI: 0.02-0.10). Tables S3-S7 in the Supplementary material presents the coefficients from the models estimated, as well as robustness checks with alternative sample definitions.

## Discussion

We found that men (women) who experienced permanent job loss in the early phase of the COVID-19 pandemic had an increase in depressive symptoms relative to before the pandemic that was more than four (two) times the increase of those without a change in their employment situation. In addition, while there were substantial differences in the likelihood of job loss across education, the increase in depressive symptoms following job loss was similar across educational levels. Interestingly, we found no evidence of the deterioration of mental health following job loss being weaker among persons with higher education. This might indicate that changes in mental health during a crisis situation do not necessarily behave in line with common beliefs that higher socioeconomic status is a protective factor. Research on mediators of job loss on mental health point to the importance of perceived financial stress and income losses [21]. Although the compensation schemes for displaced workers during COVID-19 were generous, they had upper limits. Hence, high earners experienced larger reductions in income following job loss. This could be part of the explanation for why the (absolute) increase in depressive symptoms following job loss was similar across socioeconomic status. Moreover, there could be higher stigma attached to losing work among those with higher socioeconomic status.

Our study finds a substantial difference in the increase in depressive symptoms between temporary and permanent job loss. This finding has important implications, as it suggests potentially beneficial public health effects of policy interventions that expand the allowed legal lengths of (paid) furloughs, that is, avoiding termination of work contracts during periods of sharp temporary downturns. More research on this is needed, taking into account broader differences between employment groups that experienced different forms of job loss.

Our study comes with some limitations. Our analyses are based on a select sample, both in terms of geography and demographic characteristics: MoBa is a cohort study of parents who were pregnant with a child between approximately between 2000 and 2010 and lived in Norway. Hence, the observed associations might be specific for this subgroup and could not – without strong additional assumptions – be extrapolated to those younger, older, or childless in the population. As regards the study being situated in Norway, our study results most likely provide a conservative estimate of the association between employment changes and mental health. This is because the country has one of the highest life expectancy levels in the world, highest GDP per capita, low prevalence of poverty, free and high-quality public health care, and extensive social welfare benefits [22]. Assuming that these characteristics are protective when incurring work loss, the associations we observe in our study are likely to be even stronger in countries with weaker social security systems.

## Conclusions

In a large longitudinal sample of Norwegian mothers and fathers, respondents who experienced job loss during the pandemic showed a substantially stronger increase in symptoms of depression or anxiety during the pandemic, compared to respondents with no change in their employment status. Although those with higher education had a lower risk of losing work, the negative impact of job loss on mental health was similar across educational levels.

## Supplementary Information

**Additional file 1: Table S1.** Overview of waves in MoBa. **Table S2.** Descriptives for analytical sample at Corona survey 1. **Table S3 (men):** Multivariate regression of Hopkins-scores (0-3) on time period (before vs. during the COVID-19 pandemic), employment situation during the pandemic, and controls. **Table S4 (women):** Multivariate regression of Hopkins-scores (0-3) on time period (before vs. during the COVID-19 pandemic), employment situation during the pandemic, and controls. **Table S5 (men and women):** Logit-regression of job loss (permanent or temporary; vs. no change/home office) on gender, education, and controls. Odds ratios. **Table S6 (men):** Multivariate regressions of Hopkins-scores (0-3) on time period (before vs. during the COVID-19 pandemic), job loss (permanent or temporary; vs. no change/home office) during the pandemic, education, and controls. **Table S7 (women):** Multivariate regressions of Hopkins-scores on time period (before vs. during the COVID-19 pandemic), work loss (furlough/job loss vs. no change/home office) during the pandemic, education, and controls.

## Data Availability

The consent given by the participants does not open for storage of data on an individual level in repositories or journals. Researchers who want access to data sets for replication should submit an application to datatilgang@fhi.no. Access to data sets requires approval from The Regional Committee for Medical and Health Research Ethics in Norway and an agreement with MoBa.

## Abbreviations

MoBa: Mother, Father and Child Cohort Study
GDP: Gross Domestic Product
SCL-5: Hopkins Symptom Checklist

## Footnotes

1 *Home office* was not a response option during the first pandemic data collection in 2020. The category *home office* therefore includes individuals reporting *no change* in wave 1/2020 and *home office* in waves 2/2020 and 3/2020.

2 Response options sick leave, home office and other were only added in waves 2/2020 and 3/2020. While only one option could be chosen in the first wave, multiple options could be selected in waves two and three.

3 If the father did not indicate his education in the 2^nd^ father interview, we included fathers’ education as reported by the mother (15^th^ week of pregnancy).

## References

[1] World Health Organization, O (2020). The impact of COVID-19 on mental, neurological and substance use services: results of a rapid assessment.

[2] Matthias P (2020). Mental health before and during the COVID-19 pandemic: a longitudinal probability sample survey of the UK population. The Lancet Psychiatry.

[3] Tanaka T, Okamoto S. Increase in suicide following an initial decline during the COVID-19 pandemic in Japan. Nat Hum Behav. 2021;5:229–38. 10.1038/s41562-020-01042-z 33452498

[4] Witteveen D, Velthorst E (2020). Economic hardship and mental health complaints during COVID-19. Proceedings of the National Academy of Sciences.

[5] Alstadsæter A, et al. The First Weeks of the Coronavirus Crisis: Who Got Hit, When and Why? Evidence from Norway. Natl Bur Econ Res Working Pap Ser. 2020; No.27131.

[6] Sullivan D, von Wachter T (2009). Job Displacement and Mortality: An Analysis Using Administrative Data*. Q J Econ.

[7] Browning M, Heinesen E (2012). Effect of job loss due to plant closure on mortality and hospitalization. J Health Econ.

[8] Rege M, Telle K, Votruba M (2009). THE EFFECT OF PLANT DOWNSIZING ON DISABILITY PENSION UTILIZATION. J Eur Econ Assoc.

[9] Black SE, Devereux PJ, Salvanes KG (2015). Losing Heart? The Effect of Job Displacement on Health. ILR Rev.

[10] Ruhm CJ (2003). Good times make you sick. J Health Econ.

[11] Ganson K, et al. Job Insecurity and Symptoms of Anxiety and Depression Among U.S. Young Adults During COVID-19. J Adolesc Health. 2020;68(1):53-6. 10.1016/j.jadohealth.2020.10.008 33183926

[12] Achdut, N. and T. Refaeli, Unemployment and Psychological Distress among Young People during the COVID-19 Pandemic: Psychological Resources and Risk Factors. International Journal of Environmental Research and Public Health, 2020. 17(19). 10.3390/ijerph17197163 PMC7579061 33007892

[13] Guerin R (2021). Investigating the Impact of Job Loss and Decreased Work Hours on Physical and Mental Health Outcomes Among US Adults During the COVID-19 Pandemic. J Occup Environ Med..

[14] Holingue C (2020). Mental Distress during the COVID-19 Pandemic among US Adults without a Pre-existing Mental Health Condition: Findings from American Trend Panel Survey. Prev Med.

[15] Posel D, Oyenubi A, Kollamparambil U (2021). Job loss and mental health during the COVID-19 lockdown: Evidence from South Africa. PLoS ONE.

[16] Ferry F (2021). The impact of reduced working on mental health in the early months of the COVID-19 pandemic: Results from the Understanding Society COVID-19 study. J Affect Disord..

[17] Magnus P (2016). Cohort Profile Update: The Norwegian Mother and Child Cohort Study (MoBa). Int J Epidemiol.

[18] Strand BH (2003). Measuring the mental health status of the Norwegian population: a comparison of the instruments SCL-25, SCL-10, SCL-5 and MHI-5 (SF-36). Nord J Psychiatry.

[19] Tambs K, Moum T (1993). How well can a few questionnaire items indicate anxiety and depression?. Acta Psychiatr Scand.

[20] Schmalbach B (2019). Psychometric Properties of Two Brief Versions of the Hopkins Symptom Checklist: HSCL-5 and HSCL-10. Assessment.

[21] de Miquel C (2022). The Mental Health of Employees with Job Loss and Income Loss during the COVID-19 Pandemic: The Mediating Role of Perceived Financial Stress. Int J Environ Res Public Health..

[22] Feenstra R, Inklaar R, Timmer M (2013). The Next Generation of the Penn World Table. Am Econ Rev..

