# Supplementary material for: Job loss and psychological distress during the COVID-19 pandemic: a national prospective cohort study
Source: BMC Public Health. 2023 Jul 28;23:1447. doi: 10.1186/s12889-023-16303-5 (PMC10375774; doi:10.1186/s12889-023-16303-5)
Supplement: Supplementary file 1 — Additional file 1: Table S1. Overview of waves in MoBa. Table S2. Descriptives for analytical sample at Corona survey 1. Table S3 (men): Multivariate regression of Hopkins-scores (0-3) on time period (before vs. during the COVID-19 pandemic), employment situation during the pandemic, and controls. Table S4 (women): Multivariate regression of Hopkins-scores (0-3) on time period (before vs. during the COVID-19 pandemic), employment situation during the pandemic, and controls. Table S5 (men and women): Logit-regression of job loss (permanent or temporary; vs. no change/home office) on gender, education, and controls. Odds ratios. Table S6 (men): Multivariate regressions of Hopkins-scores (0-3) on time period (before vs. during the COVID-19 pandemic), job loss (permanent or temporary; vs. no change/home office) during the pandemic, education, and controls. Table S7 (women): Multivariate regressions of Hopkins-scores on time period (before vs. during the COVID-19 pandemic), work loss (furlough/job loss vs. no change/home office) during the pandemic, education, and controls. [file 12889_2023_16303_MOESM1_ESM.docx]

**Supplementary material**

**Table of contents**

[**Table S1:** Overview of waves in MoBa 2](#_Toc135664684)

[**Table S2:** Descriptives for analytical sample at Corona survey 1 3](#_Toc135664685)

[**Table S3 (men):** Regression model and robustness checks Figure 1, Panel A 4](#_Toc135664686)

[**Table S4 (women):** Regression model and robustness checks Figure 1, Panel B 5](#_Toc135664687)

[**Table S5 (men and women):** Regression model and robustness checks Figure 1, Panel C 6](#_Toc135664688)

[**Table S6 (men):** Regression model and robustness checks Figure 1, Panel D (men). 7](#_Toc135664689)

[**Table S7 (women):** Regression model and robustness checks Figure 1, Panel D (women). 8](#_Toc135664690)

**Table S1:** Overview of waves in MoBa.

| Wave | Respondent | Time | Employment Change | Hopkins | Raw data  (MoBa) | | Analytical sample  (Figure 1, Panels A and B) | |
| --- | --- | --- | --- | --- | --- | --- | --- | --- |
|  |  |  |  |  | Person-years | Individuals | Person-years | Individuals |
| MoBa W1 | Mother | 1999-2009 |  | x | 102,150 | 86,390 | 39,410 | 32,961 |
| MoBa W5 | Mother | 2005-2014 |  | x | 41,617 | 36,823 | 19,017 | 16,875 |
| MoBa W8 | Mother | 2003-2017 |  | x | 43,616 | 37,898 | 20,704 | 17,974 |
| MoBa W1F | Father | 2000-2009 |  | x | 77,241 | 66,974 | 27,270 | 23,529 |
| MoBa W2F | Father | 2015-2018 |  | x | 29,345 | 29,306 | 13,271 | 13,271 |
| Corona-survey 1 | Both | March 2020 | x | x | 114,489 | 114,489 | 57,433 | 57,433 |
| Corona-survey 2 | Both | April 2020 | x | x | 109,870 | 109,870 | 58,603 | 58,603 |
| Corona-survey 3 | Both | May 2020 | x | x | 101,744 | 101,744 | 58,603 | 58,603 |

Note. x indicates the periods in which core variables for our study were collected.

**Table S2:** Descriptives for analytical sample at Corona survey 1.

|  | Mean/Percent | SD | N/n |
| --- | --- | --- | --- |
| Age | 47.1 | 5.2 | 57,433 |
| Female | 58.5 | - | 57,433 |
| Hopkins SCL-5 (1999-2008) | 0.21 | 0.36 | 57,433/ 116,775 |
| Hopkins SCL-5 (2020) | 0.37 | 0.46 | 57,433/ 171,574 |
| *Employment change* |  |  | 57,433 |
| Temporary job loss | 7.5 | - |  |
| Permanent job loss | 0.3 | - |  |
| Home office | 37.7 | - |  |
| No change | 54.5 | - |  |
| *Pre-COVID employment situation* |  |  | 57,433 |
| Vocational training | 0.04 | - |  |
| Public sector | 42.9 | - |  |
| Private sector | 48.2 | - |  |
| Self employed | 6.6 | - |  |
| None of the above | 7.3 | - |  |
| *Education* |  |  | 57,433 |
| Primary or secondary | 5.3 | - |  |
| Vocational training | 13.4 | - |  |
| 3 years advanced general | 11.2 | - |  |
| University up to 3 years | 34.0 | - |  |
| University 4 years and more | 32.5 | - |  |
| Missing | 3.5 | - |  |

Note. N = individuals, n=observations.

**Table S3 (men):** Multivariate regression of Hopkins-scores (0-3) on time period (before vs. during the COVID-19 pandemic), employment situation during the pandemic, and controls.

|  | M1 | M2 | M3 |
| --- | --- | --- | --- |
| 2020 | 0.12^***^ | 0.11^***^ | 0.08^***^ |
| (vs. 1999-2018) | [0.11,0.12] | [0.10,0.12] | [0.07,0.09] |
|  |  |  |  |
| Home office | 0.01^**^ | 0.02^***^ | 0.01 |
| (ref. no change) | [0.00,0.02] | [0.01,0.03] | [-0.00,0.02] |
|  |  |  |  |
| Furlough | 0.02^**^ | 0.03^***^ | 0.03^**^ |
| (ref. no change) | [0.01,0.04] | [0.02,0.05] | [0.01,0.04] |
|  |  |  |  |
| Job loss | 0.07 | 0.09 | 0.10 |
| (ref. no change) | [-0.02,0.16] | [-0.01,0.19] | [-0.02,0.23] |
|  |  |  |  |
| Home office * 2020 | 0.01^**^ | 0.02^***^ | 0.02^***^ |
|  | [0.00,0.02] | [0.01,0.03] | [0.01,0.03] |
|  |  |  |  |
| Furlough * 2020 | 0.19^***^ | 0.20^***^ | 0.19^***^ |
|  | [0.17,0.21] | [0.18,0.22] | [0.16,0.21] |
|  |  |  |  |
| Job loss * 2020 | 0.42^***^ | 0.38^***^ | 0.39^***^ |
|  | [0.29,0.54] | [0.25,0.52] | [0.19,0.59] |
|  |  |  |  |
| Persons in household (ref. 0 others) | |  |  |
| 1 | -0.06^*^ | -0.05 | -0.05 |
|  | [-0.12,-0.01] | [-0.10,0.01] | [-0.12,0.03] |
|  |  |  |  |
| 2 | -0.13^***^ | -0.08^**^ | -0.12^**^ |
|  | [-0.17,-0.08] | [-0.13,-0.03] | [-0.18,-0.05] |
|  |  |  |  |
| 3 | -0.16^***^ | -0.11^***^ | -0.14^***^ |
|  | [-0.21,-0.11] | [-0.15,-0.06] | [-0.21,-0.07] |
|  |  |  |  |
| 4 | -0.17^***^ | -0.11^***^ | -0.15^***^ |
|  | [-0.22,-0.12] | [-0.16,-0.07] | [-0.21,-0.08] |
|  |  |  |  |
| 5 or more | -0.17^***^ | -0.11^***^ | -0.15^***^ |
|  | [-0.21,-0.12] | [-0.16,-0.06] | [-0.22,-0.08] |
|  |  |  |  |
| Constant | 0.38^***^ | 0.33^**^ | 0.33^*^ |
|  | [0.19,0.56] | [0.12,0.54] | [0.02,0.64] |
| Observations | 113,040 | 108,603 | 72,874 |
| Persons | 24,486 | 23,484 | 14,287 |

95% confidence intervals in brackets. ^*^ *p* < 0.05, ^**^ *p* < 0.01, ^***^ *p* < 0.001. Models include dummy variables for age. Ages 19 and younger and 57 and older are combined into one category each to allow for better estimation (avoiding small cells).M1: Model as reported in the article. M2: Sample limited to persons who in 2020 reported pre-pandemic employment in the private or public sector, being self-employed, or being in apprenticeship. M3: Sample is limited to persons who participated in the study at least twice before (1999-2018) and at least twice during the COVID-19 pandemic (2020).

**Table S4 (women):** Multivariate regression of Hopkins-scores (0-3) on time period (before vs. during the COVID-19 pandemic), employment situation during the pandemic, and controls.

|  | M1 | M2 | M3 |
| --- | --- | --- | --- |
| 2020 | 0.20^***^ | 0.17^***^ | 0.17^***^ |
| (ref. 1999-2018) | [0.19,0.21] | [0.16,0.18] | [0.16,0.18] |
|  |  |  |  |
| Home office | -0.01^***^ | 0.01^***^ | -0.01^**^ |
| (ref. no change) | [-0.02,-0.01] | [0.01,0.02] | [-0.02,-0.01] |
|  |  |  |  |
| Furlough | -0.00 | 0.03^***^ | 0.00 |
| (ref. no change) | [-0.02,0.01] | [0.01,0.04] | [-0.01,0.02] |
|  |  |  |  |
| Job loss | 0.08^*^ | 0.12^**^ | 0.08 |
| (ref. no change) | [0.01,0.15] | [0.04,0.20] | [-0.00,0.16] |
|  |  |  |  |
| Home office * 2020 | 0.01 | 0.02^***^ | 0.01^*^ |
|  | [-0.00,0.02] | [0.01,0.03] | [0.00,0.02] |
|  |  |  |  |
| Furlough * 2020 | 0.16^***^ | 0.17^***^ | 0.15^***^ |
|  | [0.14,0.18] | [0.16,0.19] | [0.13,0.17] |
|  |  |  |  |
| Job loss * 2020 | 0.27^***^ | 0.27^***^ | 0.29^***^ |
|  | [0.17,0.36] | [0.17,0.37] | [0.18,0.41] |
|  |  |  |  |
| Persons in household (ref. 0 others) | |  |  |
| 1 | -0.09 | 0.01 | -0.09 |
|  | [-0.18,0.00] | [-0.07,0.10] | [-0.22,0.04] |
|  |  |  |  |
| 2 | -0.15^**^ | -0.02 | -0.16^*^ |
|  | [-0.24,-0.06] | [-0.11,0.07] | [-0.29,-0.03] |
|  |  |  |  |
| 3 | -0.22^***^ | -0.07 | -0.23^***^ |
|  | [-0.31,-0.13] | [-0.16,0.01] | [-0.36,-0.10] |
|  |  |  |  |
| 4 | -0.23^***^ | -0.09 | -0.25^***^ |
|  | [-0.32,-0.14] | [-0.17,0.00] | [-0.38,-0.12] |
|  |  |  |  |
| 5 or more | -0.22^***^ | -0.08 | -0.24^***^ |
|  | [-0.31,-0.13] | [-0.17,0.00] | [-0.37,-0.11] |
|  |  |  |  |
| Constant | 0.80^***^ | 0.60^***^ | 0.75^***^ |
|  | [0.66,0.93] | [0.45,0.75] | [0.57,0.94] |
| Observations | 181,271 | 165,185 | 138,116 |
| Persons | 34,496 | 31,255 | 23,461 |

95% confidence intervals in brackets. ^*^ *p* < 0.05, ^**^ *p* < 0.01, ^***^ *p* < 0.001. Models include dummy variables for age. Ages 19 and younger and 57 and older are combined into one category each to allow for better estimation (avoiding small cells). M1: Model as reported in the article. M2: Sample limited to persons who in 2020 reported pre-pandemic employment in the private or public sector, being self-employed, or being in apprenticeship. M3: Sample is limited to persons who participated in the study at least twice before (1999-2018) and at least twice during the COVID-19 pandemic (2020).

**Table S5 (men and women):** Logit-regression of job loss (permanent or temporary; vs. no change/home office) on gender, education, and controls. Odds ratios.

|  | M1 | M2 | M3 | M4 |
| --- | --- | --- | --- | --- |
| Men | 0.79^***^ | 0.76^***^ | 0.67^***^ | 0.75^***^ |
| (ref. women) | [0.72,0.86] | [0.67,0.86] | [0.61,0.74] | [0.66,0.85] |
|  |  |  |  |  |
| University | 0.29^***^ | 0.29^***^ | 0.25^***^ | 0.27^***^ |
| (ref. no university) | [0.27,0.32] | [0.27,0.32] | [0.23,0.27] | [0.24,0.30] |
|  |  |  |  |  |
| Men * university | 1.42^***^ | 1.49^***^ | 1.61^***^ | 1.61^***^ |
|  | [1.25,1.61] | [1.27,1.74] | [1.41,1.83] | [1.36,1.89] |
|  |  |  |  |  |
| Persons in household (ref. 0 others) | |  |  |  |
| 1 | 1.05 | 0.74 | 0.94 | 0.97 |
|  | [0.75,1.46] | [0.49,1.10] | [0.66,1.33] | [0.59,1.62] |
|  |  |  |  |  |
| 2 | 1.01 | 0.74 | 0.84 | 0.90 |
|  | [0.74,1.39] | [0.51,1.08] | [0.61,1.17] | [0.55,1.45] |
|  |  |  |  |  |
| 3 | 0.98 | 0.73 | 0.78 | 0.85 |
|  | [0.72,1.33] | [0.50,1.05] | [0.57,1.08] | [0.53,1.36] |
|  |  |  |  |  |
| 4 | 0.90 | 0.67^*^ | 0.72^*^ | 0.79 |
|  | [0.66,1.23] | [0.46,0.98] | [0.52,0.99] | [0.49,1.28] |
|  |  |  |  |  |
| 5 or more | 0.89 | 0.62^*^ | 0.73 | 0.73 |
|  | [0.65,1.23] | [0.42,0.91] | [0.52,1.02] | [0.45,1.19] |
|  |  |  |  |  |
| Constant | 0.20^***^ | 0.27^***^ | 0.38^**^ | 0.05^**^ |
|  | [0.10,0.37] | [0.13,0.53] | [0.19,0.73] | [0.01,0.37] |
| Observations | 56,882 | 46,594 | 52,824 | 37,440 |
| Persons | 56,882 | 46,594 | 52,824 | 37,440 |

Note: Odds ratios/exponentiated coefficients; 95% confidence intervals in brackets.
^*^ *p* < 0.05, ^**^ *p* < 0.01, ^***^ *p* < 0.001.

Models include dummy variables for age. Ages 19 and younger and 57 and older are combined into one category each to allow for better estimation (avoiding small cells). M1: Model as reported in the article. M2: Not using partner-reported education if education from men is missing. M3: Sample limited to persons who in 2020 reported pre-pandemic employment in the private or public sector, being self-employed, or being in apprenticeship. M4: Sample is limited to persons who participated in the study at least twice before (1999-2018) and at least twice during the COVID-19 pandemic (2020).

**Table S6 (men):** Multivariate regressions of Hopkins-scores (0-3) on time period (before vs. during the COVID-19 pandemic), job loss (permanent or temporary; vs. no change/home office) during the pandemic, education, and controls.

|  | M1 | M2 | M3 | M4 |
| --- | --- | --- | --- | --- |
| 2020 | 0.13^***^ | 0.09^***^ | 0.13^***^ | 0.10^***^ |
| (ref. 1999-2018) | [0.12,0.14] | [0.08,0.11] | [0.11,0.14] | [0.08,0.11] |
|  |  |  |  |  |
| Work loss | 0.02^*^ | 0.03 | 0.04^***^ | 0.03^*^ |
| (ref. no change/home office) | [0.00,0.04] | [-0.00,0.06] | [0.02,0.06] | [0.00,0.05] |
|  |  |  |  |  |
| University | -0.00 | -0.01 | 0.01 | -0.00 |
| (ref. no university) | [-0.01,0.01] | [-0.02,0.00] | [-0.00,0.01] | [-0.01,0.01] |
|  |  |  |  |  |
| University * | -0.03^***^ | -0.01^*^ | -0.02^**^ | -0.01^*^ |
| 2020 | [-0.04,-0.01] | [-0.03,-0.00] | [-0.03,-0.01] | [-0.03,-0.00] |
|  |  |  |  |  |
| Work loss * | 0.18^***^ | 0.14^***^ | 0.19^***^ | 0.15^***^ |
| 2020 | [0.14,0.21] | [0.09,0.18] | [0.15,0.22] | [0.11,0.19] |
|  |  |  |  |  |
| Work loss * | -0.01 | -0.00 | -0.02 | -0.01 |
| university | [-0.03,0.02] | [-0.04,0.03] | [-0.04,0.01] | [-0.04,0.03] |
|  |  |  |  |  |
| Work loss * | 0.03 | 0.07^*^ | 0.02 | 0.06^*^ |
| university * 2020 | [-0.02,0.07] | [0.01,0.12] | [-0.03,0.07] | [0.00,0.11] |
|  |  |  |  |  |
| Persons in household (ref. 0 others) | |  |  |  |
| 1 | -0.06^*^ | -0.06 | -0.04 | -0.05 |
|  | [-0.12,-0.01] | [-0.14,0.02] | [-0.09,0.02] | [-0.13,0.03] |
|  |  |  |  |  |
| 2 | -0.12^***^ | -0.12^***^ | -0.07^**^ | -0.12^***^ |
|  | [-0.17,-0.07] | [-0.20,-0.05] | [-0.11,-0.02] | [-0.19,-0.05] |
|  |  |  |  |  |
| 3 | -0.15^***^ | -0.14^***^ | -0.09^***^ | -0.14^***^ |
|  | [-0.20,-0.10] | [-0.22,-0.07] | [-0.14,-0.05] | [-0.21,-0.07] |
|  |  |  |  |  |
| 4 | -0.16^***^ | -0.15^***^ | -0.10^***^ | -0.15^***^ |
|  | [-0.21,-0.11] | [-0.22,-0.08] | [-0.15,-0.05] | [-0.22,-0.08] |
|  |  |  |  |  |
| 5 or more | -0.16^***^ | -0.16^***^ | -0.10^***^ | -0.15^***^ |
|  | [-0.21,-0.11] | [-0.23,-0.09] | [-0.15,-0.05] | [-0.22,-0.08] |
|  |  |  |  |  |
| Constant | 0.34^**^ | 0.36^*^ | 0.30^*^ | 0.33^*^ |
|  | [0.13,0.55] | [0.02,0.70] | [0.07,0.53] | [0.02,0.64] |
| Observations | 108,012 | 65,611 | 103,902 | 72,336 |
| Persons | 23,237 | 12,949 | 22,318 | 14,179 |

95% confidence intervals in brackets. ^*^ *p* < 0.05, ^**^ *p* < 0.01, ^***^ *p* < 0.001. Models include dummy variables for age. Ages 19 and younger and 57 and older are combined into one category each to allow for better estimation (avoiding small cells).

M1: Model as reported in the article. M2: Not using partner-reported education if education from men is missing. M3: Sample limited to persons who in 2020 reported pre-pandemic employment in the private or public sector, being self-employed, or being in apprenticeship. M4: Sample is limited to persons who participated in the study at least twice before (1999-2018) and at least twice during the COVID-19 pandemic (2020).

**Table S7 (women):** Multivariate regressions of Hopkins-scores on time period (before vs. during the COVID-19 pandemic), work loss (furlough/job loss vs. no change/home office) during the pandemic, education, and controls.

|  | M1 | M2 | M3 | M4 |
| --- | --- | --- | --- | --- |
| 2020 | 0.19^***^ | 0.19^***^ | 0.17^***^ | 0.17^***^ |
| (ref. 1999-2018) | [0.18,0.21] | [0.18,0.21] | [0.15,0.18] | [0.15,0.19] |
|  |  |  |  |  |
| Work loss | -0.02 | -0.02 | 0.01 | -0.01 |
| (ref. no change/home office) | [-0.04,0.00] | [-0.04,0.00] | [-0.01,0.04] | [-0.04,0.01] |
|  |  |  |  |  |
| University | -0.06^***^ | -0.06^***^ | -0.04^***^ | -0.05^***^ |
| (ref. no university) | [-0.07,-0.05] | [-0.07,-0.05] | [-0.05,-0.03] | [-0.06,-0.04] |
|  |  |  |  |  |
| University * | -0.01 | -0.01 | 0.01 | 0.00 |
| 2020 | [-0.02,0.01] | [-0.02,0.01] | [-0.00,0.02] | [-0.01,0.02] |
|  |  |  |  |  |
| Work loss * | 0.13^***^ | 0.13^***^ | 0.15^***^ | 0.14^***^ |
| 2020 | [0.10,0.16] | [0.10,0.16] | [0.12,0.18] | [0.10,0.17] |
|  |  |  |  |  |
| Work loss * | 0.02 | 0.02 | 0.00 | 0.02 |
| university | [-0.01,0.05] | [-0.01,0.05] | [-0.03,0.03] | [-0.01,0.05] |
|  |  |  |  |  |
| Work loss * | 0.06^**^ | 0.06^**^ | 0.04^*^ | 0.03 |
| university * 2020 | [0.02,0.10] | [0.02,0.10] | [0.00,0.08] | [-0.02,0.07] |
|  |  |  |  |  |
| Persons in household (ref. 0 others) | |  |  |  |
| 1 | -0.11^*^ | -0.11^*^ | 0.00 | -0.10 |
|  | [-0.20,-0.01] | [-0.20,-0.01] | [-0.09,0.09] | [-0.24,0.03] |
|  |  |  |  |  |
| 2 | -0.16^***^ | -0.16^***^ | -0.03 | -0.17^*^ |
|  | [-0.26,-0.07] | [-0.26,-0.07] | [-0.13,0.06] | [-0.30,-0.04] |
|  |  |  |  |  |
| 3 | -0.23^***^ | -0.23^***^ | -0.08 | -0.24^***^ |
|  | [-0.32,-0.13] | [-0.32,-0.13] | [-0.17,0.01] | [-0.37,-0.10] |
|  |  |  |  |  |
| 4 | -0.24^***^ | -0.24^***^ | -0.09^*^ | -0.25^***^ |
|  | [-0.33,-0.15] | [-0.33,-0.15] | [-0.19,-0.00] | [-0.38,-0.12] |
|  |  |  |  |  |
| 5 or more | -0.23^***^ | -0.23^***^ | -0.09^*^ | -0.25^***^ |
|  | [-0.33,-0.14] | [-0.33,-0.14] | [-0.19,-0.00] | [-0.38,-0.11] |
|  |  |  |  |  |
| Constant | 0.80^***^ | 0.80^***^ | 0.60^***^ | 0.76^***^ |
|  | [0.66,0.94] | [0.66,0.94] | [0.45,0.76] | [0.57,0.94] |
| Observations | 177,752 | 177,752 | 162,090 | 137,131 |
| Persons | 33,644 | 33,644 | 30,506 | 23,261 |

95% confidence intervals in brackets. ^*^ *p* < 0.05, ^**^ *p* < 0.01, ^***^ *p* < 0.001. Models include dummy variables for age. Ages 19 and younger and 57 and older are combined into one category each to allow for better estimation (avoiding small cells).

M1: Model as reported in the article. M2: Not using partner-reported education if education from men is missing. M3: Sample limited to persons who in 2020 reported pre-pandemic employment in the private or public sector, being self-employed, or being in apprenticeship. M4: Sample is limited to persons who participated in the study at least twice before (1999-2018) and at least twice during the COVID-19 pandemic (2020).
